# Identification of Lignan Compounds as New 6-Phosphogluconate Dehydrogenase Inhibitors for Lung Cancer

**DOI:** 10.3390/metabo13010034

**Published:** 2022-12-24

**Authors:** Gul Bushra Khan, Muhammad Qasim, Azhar Rasul, Usman Ali Ashfaq, Abdullah M. Alnuqaydan

**Affiliations:** 1 Department of Bioinformatics and Biotechnology, Faculty of Life Sciences, Government College University, Faisalabad 38000, Pakistan; 2 Department of Zoology, Faculty of Life Sciences, Government College University, Faisalabad 38000, Pakistan; 3 Department of Medical Biotechnology, College of Applied Medical Sciences, Qassim University, Buraydah 51452, Saudi Arabia

**Keywords:** in silico, docking, 6-PGD, natural inhibitors, enzymatic assay, MD simulation

## Abstract

Targeting pentose phosphate pathway (PPP) enzymes has emerged as a promising strategy to combat cancer. 6-Phosphogluconate dehydrogenase (6-PGD), the third critical enzyme of the PPP, catalyzes oxidative decarboxylation of 6-phosphogluconate (6-PG) to produce ribulose-5-phosphate (Ru-5-P) and CO2. Overexpression of 6-PGD has been reported in multiple cancers and is recognized as a potential anticancer drug target. The current study is focused on the utilization of indispensable virtual screening tools for structure-based drug discovery. During the study, 17,000 natural compounds were screened against the 3-phosphoglycerate (3-PG) binding site of 6-PGD through a molecular operating environment (MOE), which revealed 115 inhibitors with higher selectivity and binding affinity. Out of the 115 best-fit compounds within the 6-PGD binding cavity, 15 compounds were selected and optimized through stringent in silico ADMET assessment models that justified the desirable pharmacokinetic, pharmacodynamic and physicochemical profiles of 5 ligands. Further protein–ligand stability assessment through molecular dynamics (MD) simulation illustrated three potential hits, secoisolariciresinol, syringaresinol and cleomiscosin A, with stable confirmation. Moreover, 6-PGD inhibitor validation was performed by an in vitro enzymatic assay using human erythrocytes purified 6-PGD protein and A549 cell lysate protein. The results of the in vitro assays supported the in silico findings. In order to gain insight into the anticancer activity of the aforementioned compounds, they were subjected to CLC-Pred, an in silico cytotoxicity browsing tool, which proved their anticancer activity against several cancer cell lines at Pa > 0.5. Additionally, a confirmation for in silico cytotoxicity was made by 3-(4,5-dimethylthiazol-2-yl)-2,5-diphenyltetrazolium bromide (MTT) assay for commercially available hits syringaresinol and cleomiscosin A against lung cancer (A549) cells. The results demonstrated that syringaresinol has an IC_50_ value of 36.9 μg/mL, while cleomiscosin A has an IC_50_ value of 133 μg/mL. After MTT, flow cytometry analysis confirmed that compounds induced apoptosis in A549 cells in a dose-dependent manner. This study suggested that the respective lignan compounds can serve as lead candidates for lung cancer therapy via 6-PGD inhibition. Furthermore, in vivo experiments need to be conducted to confirm their efficacy.

## 1. Introduction

Cancer represents the most challenging public health burden and the leading cause of mortality faced by the world [1]. The emerging cancer hallmarks include deregulating cellular energetics, avoiding immune destruction, tumor-promoting inflammation, genome instability and mutation, and tumor metabolism [2]. In the context of cancer metabolism, glucose metabolism has captured a great deal of attention in cancer research in the past few decades [3]. The Warburg effect, which is marked by immense glucose uptake and lactate production even in the presence of sufficient oxygen supply and fully functional mitochondria, is acknowledged as a major cancer hallmark and an essential contributor to metabolic rewiring [4,5].

Metabolic reprogramming-induced alterations support tumor cells by accomplishing crucial demands, such as macromolecular biosynthesis, high energy production and redox homeostasis maintenance [6,7]. Therefore, altered glucose metabolism is not just confined to glycolysis but also extended to other glucose-utilizing metabolic pathways, such as the pentose phosphate pathway (PPP) [4]. The PPP, also termed the hexose monophosphate shunt (HMP) or phosphogluconate pathway, is a glycolysis parallel metabolic pathway [8] that shunts off glycolysis after the first step and generates pentose phosphate for nucleic acid and NADPH for fatty acid synthesis and cell survival in stress environments [9,10]. The PPP communicates several important features to cancer cells, such as increased tumor growth, escaped apoptosis and supporting angiogenesis and metastasis [8].

Studies have shown that PPP flux can be regulated either directly or indirectly to ameliorate cancer cell growth and viability [11]. It has been suggested by previous research that increased PPP flux in tumor cells serves as a line of distinction for normal cells [9,10], which is followed by overexpression of PPP enzymes (G6PD and 6-PGD, the first and third enzymes that link glycolysis to PPP by anabolic biosynthesis and NADPH production to maintain redox homeostasis in mammalian cells) in several tumors; therefore, PPP enzymes have been characterized as potential targets for cancer treatment and diagnosis recently. 6-Phosphogluconate dehydrogenase (6-PGD), a critical enzyme of the PPP, is involved in the oxidative decarboxylation of 6-phosphogluconate (6-PG) to produce ribulose-5-phosphate (Ru-5-P) and CO2 [8,12]. 6-PGD overexpression has been demonstrated in several cancers, such as thyroid, hepatic, breast, colon, ovarian and lung [12,13,14], and this deregulated expression promotes tumorigenesis [12]. Activation of 6-PGD is involved in anabolic biosynthesis, redox homeostasis and glycolysis, which supply the metabolic advantage to enhance cancer cell survival and proliferation [15]. Inhibition of 6PGD has been accounted to suppress tumor growth and induce cell death (by xenograft experimentation in nude mice with H1299, and K562 cells containing inducible 6-PGD shRNA construct in the presence of doxycycline) and sensitize cancer cells to chemotherapy (by exposing 6-PGD-depleted breast cancer cells and an in vivo xenograft mouse model to combined treatment with physion and paclitaxel) [16].

In the past few years, computer-aided drug design (CADD) has gained a lot of momentum as a drug discovery pipeline in the academic world and in the pharmaceutical industry [17]. Molecular modeling is an indispensable tool in the drug discovery toolbox to predict favorable ligand–receptor orientations [18,19]. The importance of using in silico methods of phenotypic screening instead of the experimental in vivo screening of anticancer agents is to reduce the time, cost and testing of animals for assessing a variety of anticancer agents [20]. The current study investigated molecular docking to explore the phytochemical database to obtain five potential candidates with good binding site interactions and other drug-likeness parameters. Docking orientations of protein–ligand complexes were further subjected to time-dependent motions through molecular dynamics (MD) simulation, which led to the identification of three lignan compounds. Selected compounds were then validated by enzyme activity assay against 6-PGD. Cytotoxicity investigation of commercially available compounds syringaresinol and cleomiscosin A by MTT assay found those compounds as potential therapeutic agents. Thus, the present study disclosed lignan compounds as 6-PGD inhibitors that can be used as anti-lung cancer agents with desirable safety profiles.

## 2. Materials and Methods

### 2.1. Targeted Protein Structure Retrieval and Refinement

The three-dimensional (3D) structure of 6-phosphogluconate dehydrogenase co-crystallized with 3-phosphoglyceric acid (3-PG) was retrieved through the Protein Data Bank (PDB) with respective PDB ID 4GWK, having a resolution of 1.58 Å. In the molecular operating environment (MOE) (2009.10), this 3D structure was edited as a co-crystallized ligand with water molecule removal by the addition of partial charges via 3D protonation and energy minimization via the MMFF94x force field, keeping the gradient as 0.05. The minimized structure was considered as a receptor to perform the docking studies [21].

### 2.2. Phytochemical Database Preparation

A phytochemical database comprising 17,000 compounds was prepared by retrieving the structures in .mol, .mol2 and .sdf formats from MAPS [20], PubChem [22], Zinc [23], MPD3 [24] and ChEMBL [25]. All ligand structures were optimized via partial charge addition and energy minimization using the MMFF94x force field [26].

### 2.3. Active Site Prediction and Molecular Docking

The MOE pocket finder tool was used to predict potential docking sites, and a site with significant binding residues was selected in order to find the interacting residues involved in contact with 6-PGD [27]. Molecular docking was performed with MOE-Dock by scanning the ready-to-dock phytochemical libraries against the active pocket residues of 6-PGD. 3-PG (PubChem ID: 724), a natural inhibitor of 6-PGD [27], was also test-docked as a reference ligand. Parameters adjusted for docking include: ligand placement by default parameters using a triangle matcher algorithm with 1000 returned poses; rescoring was performed by the London dG scoring function; retain was set to 10; refinement was performed by the force field algorithm to calculate the final energy in GBVI. The MOE program validates the accurate ligand conformation to obtain the energy-minimized structure. The LigX tool of MOE was used to analyze the 2D plots and enable a clear view of receptor–ligand interactions [21,28].

### 2.4. In Silico Drug Scan and ADMET Profiling

Further selection of the best-docked phytochemicals was made on the basis of Lipinski’s rule of five (Ro5) [29], and compounds that violated any Ro5 were eliminated from the study. This step was carried out using the Molinspiration server by entering smiles [30]. To compute the detailed drug-likeness profiles, candidate compounds were exposed to SwissADME software (Daina et al. 2017), pkCSM [30] and admetSAR in silico drugability assessment models. Calculating ADMET properties such as absorption, distribution, metabolism, excretion and toxicity is an essential hint of a drug candidate’s fate, its toxicity level in the human body and also its behavior [31].

### 2.5. Molecular Dynamics Simulation

Molecular dynamics (MD) simulation was executed by the Desmond v3.6 molecular dynamics program according to a previously reported method [32]. In brief, a boundary was prepared with an orthorhombic-shaped box, and a TIP3P solvent model was applied. System neutralization was achieved by the addition of Na+ salt through the OPLS-2005 force field. Protein–ligand minimization was carried out by a hybrid method of steepest descent and LBFGS algorithms. The MD simulation was conducted at 100 ns.

### 2.6. In Vitro Enzymatic Assays

#### 2.6.1. 6-PGD Purification Using Human Erythrocytes

The enzyme (6-PGD) was purified through a single chromatographic step based on a previously reported protocol [33]. Hemolysate was subjected to a 2,5-ADP Sepharose 4B affinity chromatography column. Subsequently, column washing was performed with 30 mL of 50 mM potassium phosphate buffer (including 1 mM EDTA, 1 mM DTT and 80 mM KCl at pH 7.35) until the final absorbance difference of 0.05 was achieved. Then, 6-PGD elution was performed with 20 mL of 80 mM potassium phosphate + 80 mM KCl + 5 mM NADP+ + 1 mM EDTA at pH 7.85. Eluted protein was monitored by enzyme activity measurement. Further, active enzyme tubes were combined and dialyzed in potassium acetate and potassium phosphate buffer (50 mM potassium acetate + 50 mM potassium phosphate).

#### 2.6.2. Protein Determination and Purity Check

Protein concentration estimation was performed according to Bradford’s method considering bovine serum albumin as the standard [34]. Enzyme purity was monitored after 6-PGD purification under denaturing conditions [33].

#### 2.6.3. In Vitro Effect of Lignan Compounds Using Erythrocytes Purified 6-PGD

To investigate the effect of lignans (syringaresinol and cleomiscosin A) on 6-PGD, different concentrations (20, 40, 80, 100 and 200 µg/mL) of both compounds were added to the reaction mixture, and then enzymatic activity was determined. All measurements were performed in triplicates. The reaction mixture without the addition of compounds was considered as the control, which showed 100% activity.

#### 2.6.4. Enzyme Activity Assay Using A549 Cell Lysate Protein

Freshly cell-cultured plates were washed with phosphate-buffered saline and lysed with lysis buffer (20 mM Tris-HCl at pH 7.5, 1 mM EDTA, 1 mM dithiothreitol 0.02%) triton X-100, 0.02%) and sodium deoxycholate) and with cocktail inhibitors (protease and phosphatase). After scrapping, cell lysates were sonicated and then centrifuged (at 12,000× *g*) for 20 min at 4 °C. Next, the supernatant was separated from the sample and was immediately used to measure the enzyme activity of 6-PGD with the help of an ELISA plate reader. Specific enzyme activity was measured by monitoring an increase or decrease in absorbance at 340 nm due to NAD(P)H. Sample enzyme activity was normalized to the total protein content of the sample that was determined by BCA assay at 550 nm [35].

#### 2.6.5. 6-Phosphogluconate Dehydrogenase (6-PGD)

To determine the specific activity of 6PGD, the sample was added to a cuvette that contained 0.5 mM NADP+ 50 mM Tris-HCl (including 0.2 mM MgCl_2_) at 37 °C with pH 7.6. The reaction was triggered by adding the substrate (6PG), by keeping the final concentration of 2 mM [35].

### 2.7. Cell Line Cytotoxicity Prediction

Cell line cytotoxicity predictor (CLC-Pred) is a validated in silico cytotoxicity evaluation tool. This server browses the cytotoxic potential of desired compounds on the basis of the structural formula [36]. The data of the compound to predict cytotoxicity were uploaded in smiles format. The calculated output was presented in the form of activation probability (Pa) and inactivation probability (Pi) values.

### 2.8. Cell Culture

Human lung adenocarcinoma epithelial cells (A549) were cultured in Dulbecco’s Modified Eagle’s Medium (DMEM) with 10% fetal bovine serum (FBS) and 100 μg/mL of antibiotic, streptomycin, and these cells were maintained at 37 °C temperature with 5% CO_2_ in a humidified environment [37].

### 2.9. MTT Cytotoxicity Assay

Cell cytotoxicity of final hits was evaluated by 3-(4,5-dimethylthiazol-2-yl)-2,5-diphenyltetrazolium bromide (MTT) assay. In this method, A549 cancer cells were cultured in 96-well plates. Afterwards, different concentrations of compounds were added to cultured cells and incubated for 48 h. After 48 h, 10 μL of MTT reagent (5 mg/mL) was added to the cells, and cells were again kept for 4 h at 37 °C. In the next step, 150 μL of DMSO was added to the preincubated cells in order to dissolve the formazan product. Subsequently, optical density was measured at 570 nm, utilizing a microplate reader [37]. Absorbance of controlled and treated cells was calculated by the following equation.

I% = [A570 (control) − A570 (treated)]/A570 (control) × 100.

### 2.10. Flow Cytometric Analysis for Apoptosis

To detect apoptosis in A549 cells, annexin V-FITC/PI was used. In brief, A549 cells were harvested in 6-well plates and kept overnight for attachment. Cells were treated with 25, 50, 100 and 200 μM of syringaresinol and cleomiscosin A for 24 h. After cell collection, they were further washed and resuspended in PBS. To determine the apoptosis, double-staining annexin V-FITC and PI was performed using the annexin V-FITC apoptosis kit (Beyotime Biotechnology Shanghai, China). Flow cytometric analysis was carried out immediately after the staining. Cell Quest software was utilized for sample data acquisition and subsequent analysis [38].

### 2.11. Statistical Analysis

Study data were expressed as the mean standard deviation of experiments that were performed in triplicates. Data analysis was performed by using GraphPad Prism and other statistical tests, including analysis of variance or ANOVA, using SPSS software, and the significance value was set at *p* ≤ 0.05 [39].

## 3. Results

### 3.1. Database Screening and Docking Study

**Figure 1:**
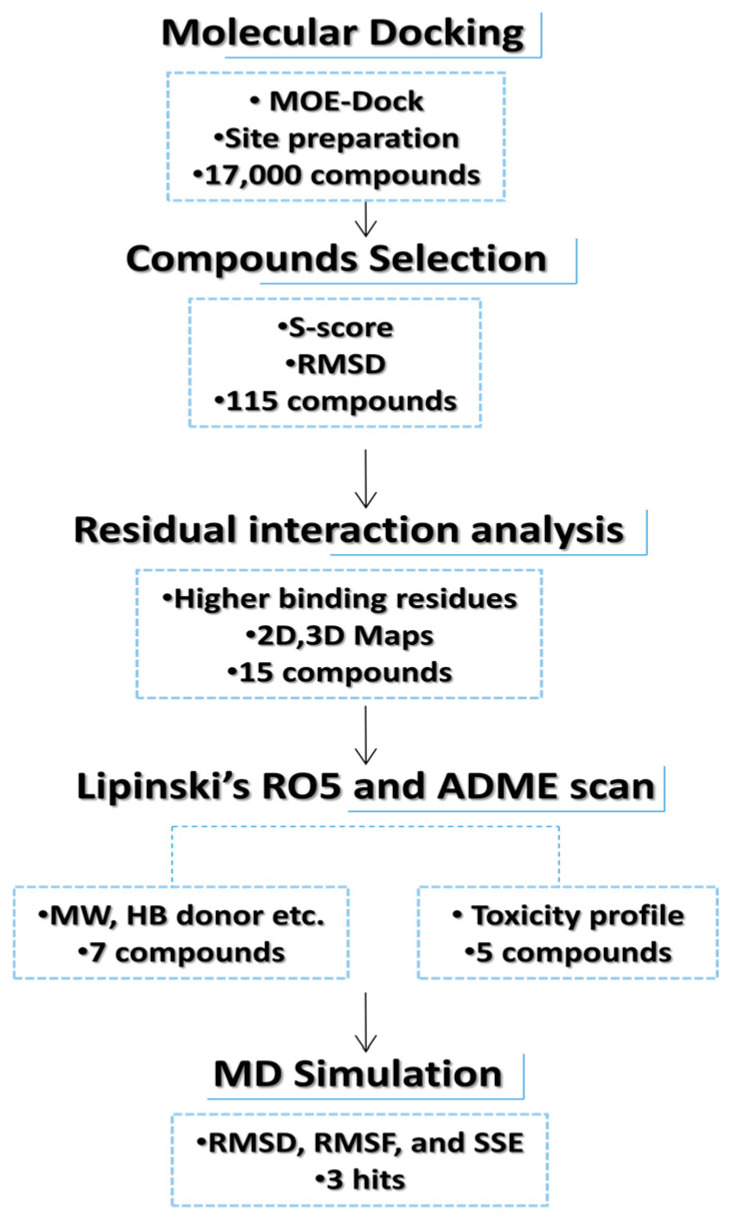
Flowchart representation of hit selection for in silico phase.

**Figure 2:**
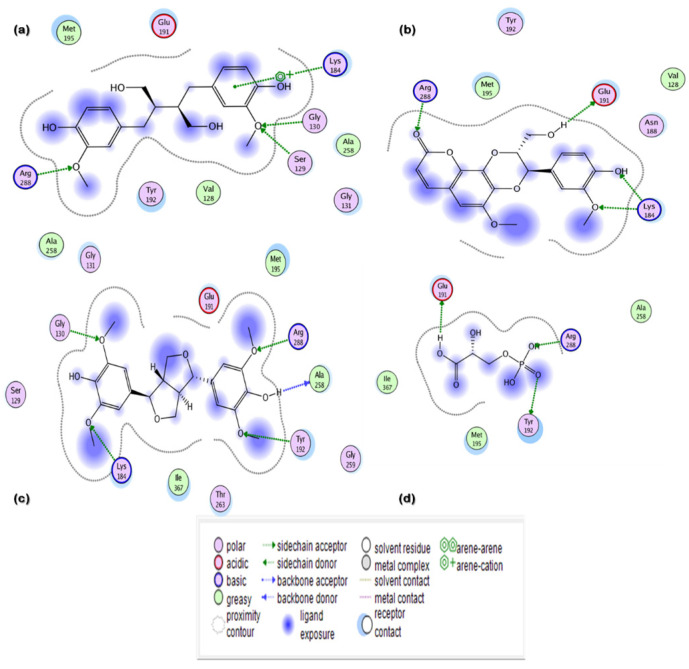
Two-dimensional (2D) interaction maps of hits with potential active site amino acid residues: (**a**) secoisolariciresinol; (**b**) syringaresinol; (**c**) cleomiscosin A; (**d**) 3-PG.

**Figure 3:**
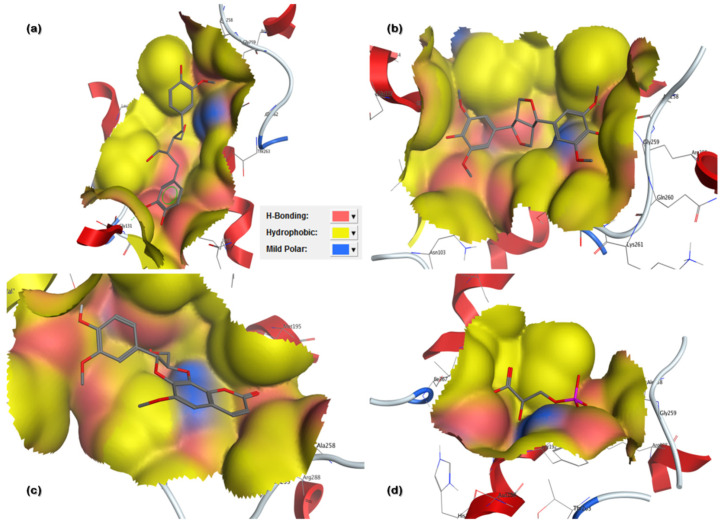
Three-dimensional (3D) binding pattern maps of hits in 6-PGD binding site representing H-bond (pink), hydrophobic interaction (yellow) and mild polarity (blue): (**a**) secoisolariciresinol and 6-PGD forming arene-cation and side-chain hydrogen bonds; (**b**) syringaresinol forming side-chain hydrogen bond acceptor and donor; (**c**) cleomiscosin A forming side-chain hydrogen bond acceptor and donor; (**d**) 3-PG forming side-chain hydrogen bond acceptor and donor.

In the current in silico experiment, a library of 17,000 phytochemicals along with 3-PG was screened against 6-PGD to predict the best binding modes (Figure 1). The docked compounds were ranked on the basis of a stringent filter that accounts for maximum habitation of binding pocket residues with the lowest Gibbs free energy, hydrogen bonding and noncovalent interactions strength, which are collectively represented as the S-score. The docking score is used to predict the binding affinity of a particular program, while a smaller root mean square deviation (RMSD) value indicates high similarity or best orientation between two structures. Therefore, 115 potential compounds were selected on the basis of the minimum S-score (greater than −10) and RMSD < 2. Further selection was made on the basis of higher binding site residues (2 or more) occupied by the ligand (Table 1) revealing 15 compounds, out of which 5 phytochemicals, secoisolariciresinol, syringaresinol, cleomiscosin A, tubulosine and terrestriamide, were selected as potential candidates. Secoisolariciresinol showed the highest binding score (−12.9507), with an RMSD value of 1.4987. The rest of the compounds showed smaller docking scores (−10.3479, −10.3156, −10.0725 and −10.0456) than secoisolariciresinol, with RMSD values of 1.6195, 1.0467, 1.7187 and 1.7236 for syringaresinol, cleomiscosin A, tubulosine and terrestriamide, respectively. All hits interacted with Lys194, Tyr192/Arg288 or both Tyr192 and Arg288. These interactions were followed by additional residues such as Gly130 in the case of secoisolariciresinol and syringaresinol, Glu191 in cleomiscosin A and Ala258 in syringaresinol. These finalized phytochemicals established different bonding interactions with 6-PGD, as follows: secoisolariciresinol formed arene-cation and side-chain hydrogen acceptor interactions, syringaresinol formed side-chain acceptor and backbone hydrogen donor interactions, cleomiscosin A formed side-chain hydrogen acceptor and donor interactions, and tubulosine and terrestriamid formed side-chain hydrogen acceptor interactions with 6-PGD. Two-dimensional (2D) ligand–receptor residue interactions and 3D interaction maps of potential compounds in the active site are shown in Figure 2 and Figure 3, respectively. It was observed in the docking results that docking poses in the scoring field (S) were ranked on the basis of binding energy calculation. Binding energy estimation of hits based on the generalized Born volume integral (GBVI) model exhibited a range of −26.086 to −18.455 kcal/mol. Docking simulations of 3-PG (direct inhibitor of 6-PGD) showed interactions with Tyr192, Arg288 and Glu191 residues of the active pocket, with a docking score and RMSD value of −8.4342 and 1.3230, respectively. The binding energy was recorded at −18.989 kcal/mol.

### 3.2. ADME Predictions and Toxicity Scan

**Figure 4:**
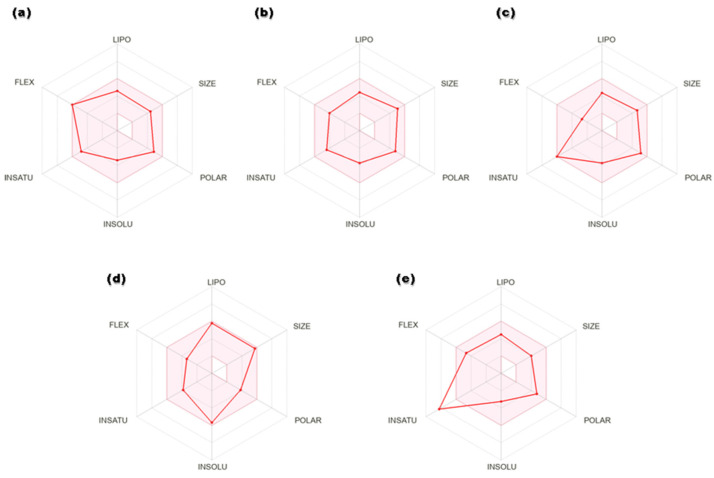
Radar plots of hits: (**a**) secoisolariciresinol, (**b**) syringaresinol, (**c**) cleomiscosin A, (**d**) tubulosine and (**e**) terrestriamide. Colored area represents suitable oral bioavailability space. Pink color: suitable bioavailability area; red color: compound bioavailability area.

Drug-like properties of the proposed 6-PGD inhibitors were calculated by the Molinspiration server available online. The selected candidates exhibited no violations of Lipinski’s rule of five, i.e., molecular weight (>500), nHB donor (>5), nHB acceptor (>10), logP (>5.8), nrotatable bonds (nRB) (>8) and polar surface area (PSA) (>140) (Table 2). The detailed pharmacokinetic and pharmacodynamic profiles of potential ligands were inspected through SwissADME, pkCSM and AdmetSAR. Solubility, an important factor of a drug, is linked with its absorption. A lesser value indicates higher solubility and good absorption, and the selected compounds showed high gastrointestinal absorption. In addition, hit compounds indicated suitable oral bioavailability, which was illustrated in terms of radar plots (Figure 4). Four compounds, secoisolariciresinol, syringaresinol, cleomiscosin A and tubulosine, showed bioavailability within the suitable zone, while terrestriamide deviated from the trend by crossing the suitable boundary. Gastrointestinal absorption analysis suggested that all five compounds possess high absorption by the gastrointestinal tract after their oral intake. After circulation, consistent distribution of the drug to every tissue assures its good efficacy. The distribution parameter describes that all compounds possess localization to mitochondria, which is one of the subcellular drug binding targets, but distribution to the blood–brain barrier is confined to tubulosine only in terms of brain tumors. Most of the hits (except tubulosine) are noninhibitors and nonsubstrate of P-glycoprotein, facilitating drug efflux from the cell. Cytochrome p450 is involved in xenobiotic compounds’ metabolism, which is used to determine the drug concentration inside the cell. Metabolism prediction of hits for cytochrome P450 inhibition indicated that secoisolariciresinol acted as CYP1A2 and CYP2C19, syringaresinol as CYP2C19, CYP3A4 and CYP2C9, tubulosine as CYP2D6, and terrestriamide as a CYP3A4 inhibitor of cytochrome isoforms, while no inhibitory effects were presented by cleomiscosin A. Physiological activation of a drug is described in terms of the bioavailability score, and all compounds showed moderate activity with a 0.5 score. The best hits did not show toxicity (AMES and hepatic) and did not pose skin irritancy and the risk of carcinogenicity. Total clearance represents the elimination of a compound from the body as a result of liver metabolism or kidney excretion. Synthetic accessibility assessed the ease of compound synthesis, and shortlisted compounds showed it is easy (more for terresriamide) to synthesize all these compounds. Detailed ADMET properties of shortlisted phytochemicals are presented in Table 3.

### 3.3. Molecular Dynamics (MD) Simulations

Molecular dynamics (MD) is an in silico approach that is utilized to model the time-dependent motions of protein–ligand complexes [40]. Validation of the best ligand poses was performed by dynamics simulation. MD simulation was analyzed for root mean square fluctuation (RMSF), solvent accessible surface area (SASA), hydrogen bond interaction and root mean square deviation (RMSD).

#### 3.3.1. RMSD and RMSF

**Figure 5:**
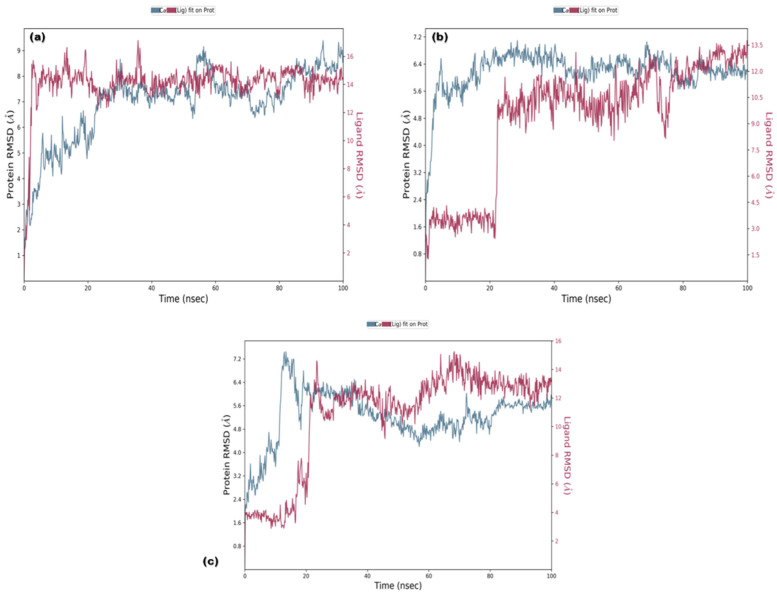
Root mean square deviation plots of docked complexes ((**a**) secoisolariciresinol, (**b**) syringaresinol and (**c**) cleomiscosin A) representing protein evolution (*y*-axis) during 100 ns MD simulations.

**Figure 6:**
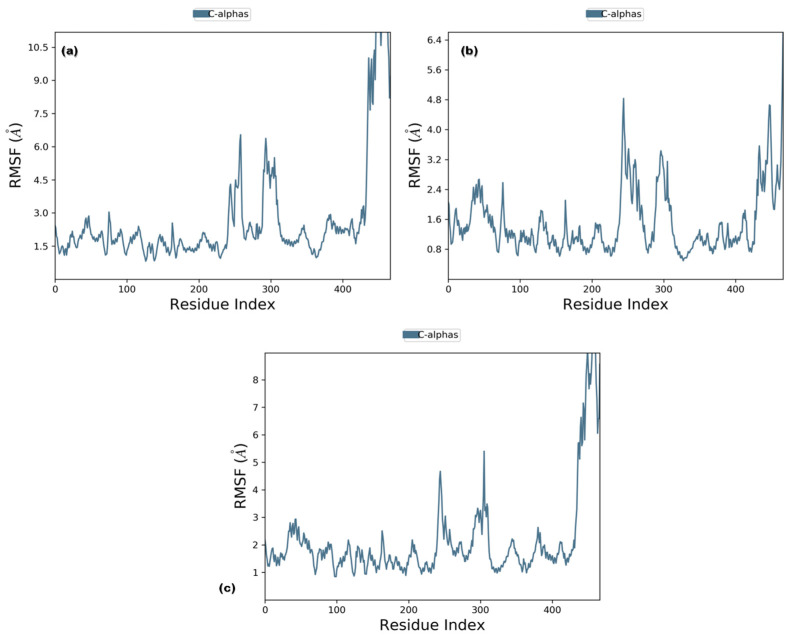
Root mean square fluctuation plots of 6PGD with candidate complexes ((**a**) secoisolariciresinol, (**b**) syringaresinol and (**c**) cleomiscosin A) representing local changes during 100 ns MD simulations.

RMSD analysis of the selected ligands suggested that the simulations were in equilibrium and deviations were within the acceptable range. This indicated that the protein did not endure large conformational changes. The Cα atoms for secoisolariciresinol showed minimal deviation within the first 5 ns, while cleomiscosin A and syringaresinol presented more deviations during the initial 20 ns before attaining equilibrium, which was achieved after 20 ns (Figure 5). The flexibility of complexes was investigated through RMSF in the function of time. The protein residual flexibility investigation revealed the highest peaks in the loop region at the 260 and 300 residues of the protein, sited away from the binding pocket residues (Figure 6). These RMSF and RMSD results are indications of the compound’s stability during simulation.

#### 3.3.2. Ligand Contacts and Interactions

**Figure 7:**
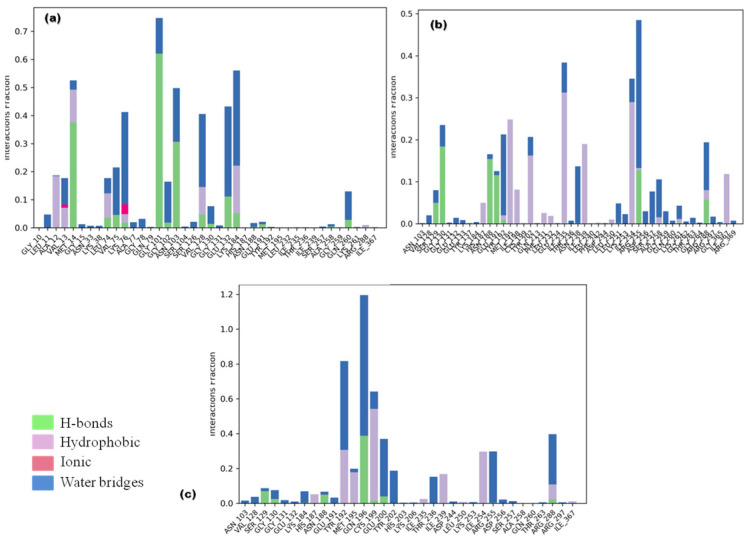
Protein–ligand interaction types between 6-PGD and candidate compounds ((**a**) secoisolariciresinol, (**b**) syringaresinol and (**c**) cleomiscosin A) during the simulation. Green color: hydrogen bonds; purple color: hydrophobic; pink color: ionic; blue color: water bridges.

Ligand fraction and contact timeline representation of candidate ligands represented a total of 34 to 47 contacts with protein residues secoisolariciresinol, cleomiscosin A and syringaresinol, respectively. The majority of hydrophobic interactions were made in the simulation trajectory of 2–55%, and hydrogen bonds were formed with Gly101, Lys184, Asn188, Arg188, Tyr192, Gly130, Arg255, Glu191 and Ser129 during 2–65% of simulation time (Figure 7).

#### 3.3.3. Protein Secondary Structure Elements (SSE)

**Figure 8:**
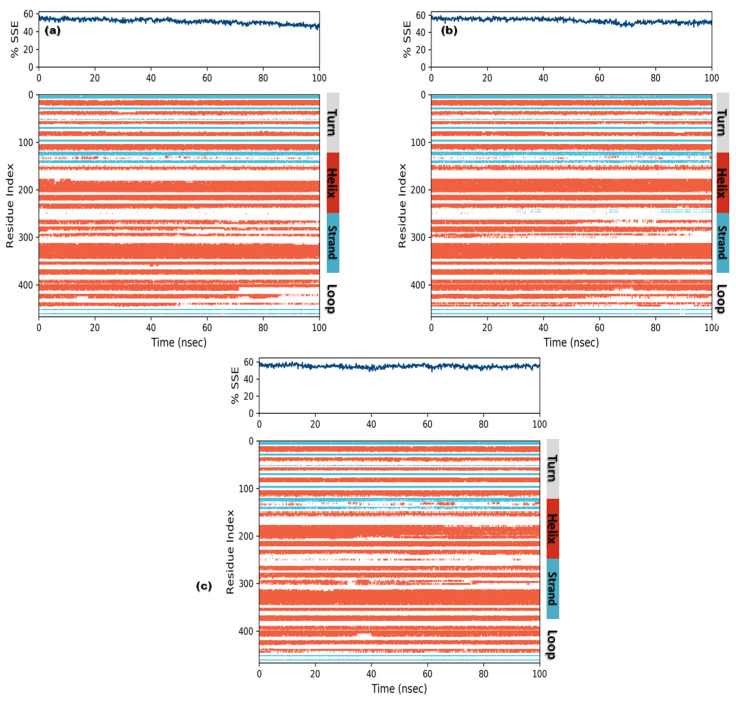
Secondary structures element composition frames for each trajectory (upper graph) and each residual SSE (lower graph) during the course of MD simulations for 3 hits ((**a**) secoisolariciresinol, (**b**) syringaresinol and (**c**) cleomiscosin A).

In order to understand conformational changes during the simulation trajectory, protein SSE elements were monitored. All complexes showed a secondary structure in terms of α-helices and β-strands. Residual index plot distribution presented 43.5%, 45.7% and 47.3% α-helices for secoisolariciresinol, cleomiscosin A and syringaresinol, which reveal the good stability of bound systems. The juncture shared by the β-strands for candidate complexes was 7.90%, 7.82% and 7.62% for secoisolariciresinol, cleomiscosin A and syringaresinol, respectively (Figure 8).

### 3.4. Enzymatic Assay Using Human Erythrocyte Purified 6-PGD

**Figure 9:**
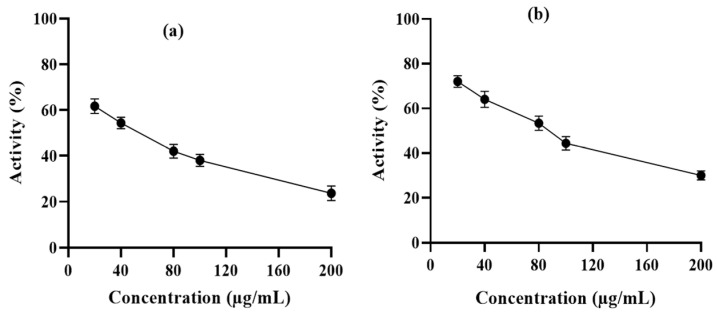
6-PGD enzyme inhibition activity isolated by erythrocytes in the presence of (**a**) syringaresinol and (**b**) cleomiscosin A at various concentrations (20, 40, 80, 100 and 200 µg/mL).

6-PGD purification was performed by using a single-step affinity chromatography method with the 2,5-ADP Sepharose 4B affinity chromatography column. In vitro enzyme activity assay was performed to determine the inhibitory effects of syringaresinol and cleomiscosin A on purified 6-PGD. This showed a dose-dependent decrease in enzyme activity with an increase in compound concentrations. 6-PGD enzymatic activity was reduced up to 76.34% by syringaresinol and 70% by cleomiscosin A at 200 µg/mL concentration (Figure 9).

### 3.5. Syringaresinol and Cleomiscosin A Are 6-PGD Inhibitors

**Figure 10:**
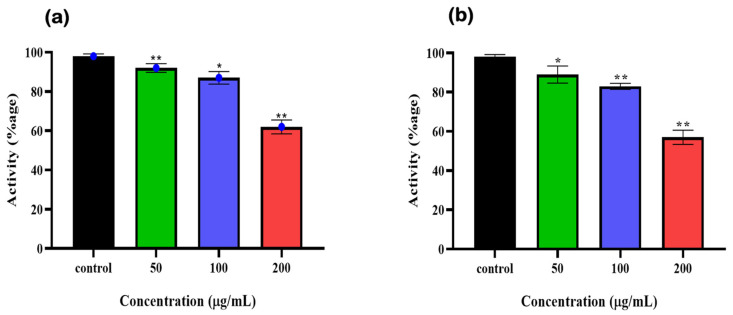
6-PGD enzyme activity purified from fresh A549 cells post treatment of (**a**) syringaresinol and (**b**) cleomiscosin A at various concentrations (50, 100 and 200 µg/mL), whereas * *p* < 0.005 and ** *p* < 0.001.

To further validate the 6-PGD inhibition by syringaresinol and cleomiscosin A (inhibitors), A549 cell (freshly cultured) lysate protein based enzymatic assay was performed. Earlier, A549 cells were treated with different concentrations of syringaresinol and cleomiscosin A. Then, enzymatic activity was measured immediately after cell scrapping, which showed dose-dependent inhibitory effects of both compounds against 6-PGD. We also observed that at a concentration of 200 µg/mL treatment, both compounds significantly inhibited 6-PGD in A549 cells as compared to control (Figure 10).

### 3.6. Cytotoxicity Prediction

The cytotoxic potential of targeted hits was presented in terms of their Pa and Pi values against cancer cell lines. According to these data, shortlisted phytocompounds were found active against several cancer cell lines at Pa > 0.5. Compounds such as cleomiscosin A and syringaresinol presented the most significant results at a higher cut-off value of Pa > 0.5, while secoisolariciresinol showed activation probability at a cut-off value of Pa > Pi for the A549 cell line. A higher activation probability (Pa) score shows more probability of a compound being cytotoxic, whereas a Pi score indicates inactivation probability. The Pa values of all compounds against the mentioned cell lines are considerably higher than the Pi values; therefore, this output can be considered as probable cytotoxicity activities for these compounds, and the (Pa, Pi) values of shortlisted compounds are mentioned in Table 4.

### 3.7. Cytotoxicity Assessment of Final Hits

**Figure 11:**
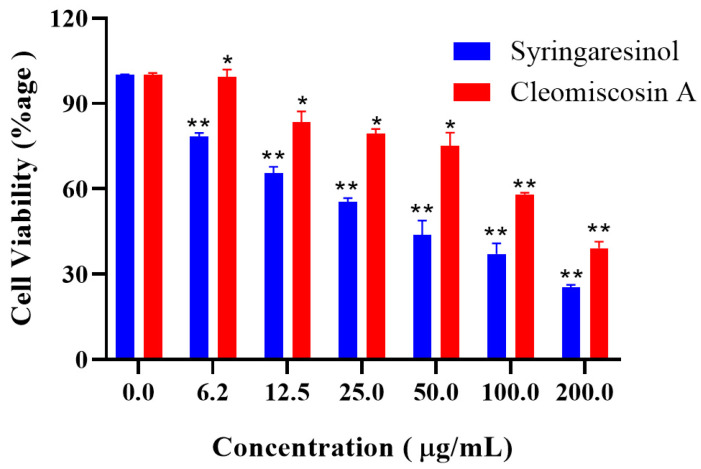
Cytotoxicity evaluation of syringaresinol and cleomiscosin A by MTT assay. Results are given as percentage activities as compared to control (untreated cells), and data are represented as mean ± SD values of experiments carried out in triplicates, where * *p* < 0.005 and ** *p* < 0.001.

Next to in silico cytotoxicity prediction of three hits, two market-available and easy-to-access compounds, syringaresinol and cleomiscosin A, were purchased to determine their anticancer ability against 6-PGD for the lung cancer (A549) cell line. The results obtained by MTT assay revealed that both compounds hold cytotoxic activity against lung cancer (Figure 11).

### 3.8. Flow Cytometry for Apoptosis

**Figure 12:**
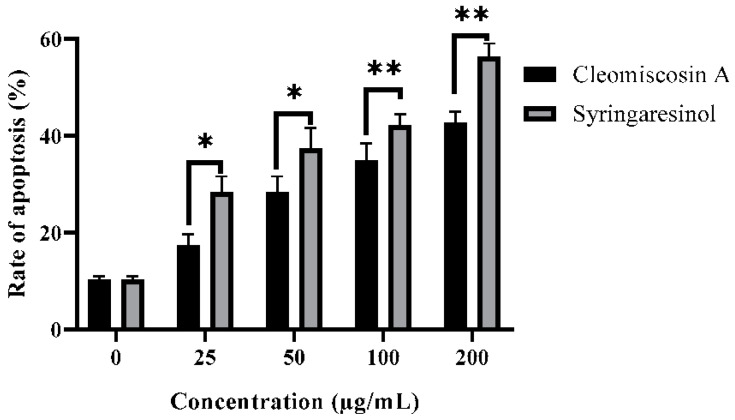
Apoptosis rates in A549 cells with or without treatment (control) by flow cytometry analysis. Cells were treated with syringaresinol and cleomiscosin A for 24 h. Data are represented as mean ± SD values of three independent experiments, where * *p* < 0.005 and ** *p* < 0.001.

In this study, we further investigated that syringaresinol and cleomiscosin A inhibited cell growth in A549 cells via apoptosis induction. Apoptosis induced by syringaresinol and cleomiscosin A was studied by flow cytometric analysis. For this purpose, cells were grown in 12-well plates, incubated without compounds (control) and with compounds and then collected in centrifuged tubes for staining with annexin V-FITC and PI double staining according to the process mentioned in the Materials and Methods section (Section 2). The results revealed the rates of apoptosis were 28.34 ± 3.25% and 17.34 ± 2.28% at 25 μM, 37.34 ± 4.25% and 28.35 ± 3.27% at 50 μM, 42.16 ± 2.26% and 34.86 ± 3.56% at 100 μM, and 56.28 ± 2.76% and 42.65 ± 2.37% at 200 μM, and 10.23 ± 0.72% for the control group in comparison to treated cells for syringaresinol and cleomiscosin A, respectively (Figure 12).

## 4. Discussion

Medicinal chemists have to face hefty issues regarding traditional drug design methods, but in silico approaches, especially molecular docking, offer efficient and time-saving strategies to design novel therapeutic agents to combat human diseases [41]. Molecular docking falls in the structure-based drug design (SBDD) category of virtual screening, which allows predicting the behavior of small molecular scaffolds in the active pocket of the receptor [42].

Phytochemicals and their derivatives present potential treatment efficacy and fewer side effects in cancer patients [43]. Therefore, in the current study, a large library of plant-derived natural compounds was subjected to the SBDD approach for the selection of potent inhibitors against 6-PGD, which identified five potential candidates, including secoisolariciresinol, syringaresinol, cleomiscosin A, tubulosine and terrestriamide, with a free binding energy range of −26.086 to −18.455 kcal/mol. According to the literature, these compounds have been reported to hold several bioactivities, such as tubulosine, which has been attributed as an anticancer and antiplasmodic agent [44,45], terresriamide as an hepatoprotective and antinitric oxide production activity compound [46,47], secoisolariciresinol and syringaresinol as good antioxidant and antimicrobial agents [48,49,50,51], and cleomiscosin A as an antioxidant as well as vasorelaxant for rat aortic ring [52,53].

Few studies have been performed previously to identify 6-PGD potent inhibitors. A study by Bayindir et al. [54] identified N-benzoylindole and its derivatives as 6-PGD inhibitors. A recent study based on rhodanines scrutinized their inhibition potential on G6PD and 6-PGD. This experiment performed a docking study using Schrodinger Maestro 12.0 on the 4GWK receptor with 3-PG and demonstrated an RMSD score of 1.532 Å. In comparison to this study, 3-PG scored a lower RMSD (1.3230 Å), and the RMSD hits were in the range of 1.0467–1.7236 Å. Rhodanine experimentation illustrated 3-amino-2-thioxothiazolidin-4-one (3-NH2-Rh) and (E)-3-((4-nitrobenzylidene)amino)-2-thioxothiazolidin-4-one as potential compounds, with docking scores of −5.234 and −5.071, respectively. In our study, the docking score is in the range of −12.9507 to −10.0456 for potential candidates. Residual interaction exhibited V128 and K184 for 3-NH2-Rh and Q130, Q131 and N188 for the second compound as significant [37], while the current study recognized Y192, R288, G130 and Q191 as potential interacting residues along with K184.

Drug efficacy and safety are primary goals to find a new drug that is helpful not only for combating diseases but also to avoid adverse effects [55]. In silico screening has played a highly significant role in the field of drug discovery by offering effective means to investigate diverse pharmacokinetic properties [55]. Docking studies selected seven potential ligands with minimum docking scores, low RMSDs and more binding site residual interactions. These compounds were then subjected to ADMET and toxicity evaluations, to exclude compounds with toxic and adverse ADMET properties that could lead to termination of experimental investigations and casue model animals to suffer during further confirmatory trials. Compounds that violated any of Lipinski’s rule of five (RO5), ideal criteria to assess an orally active drug and showed AMES toxicity and carcinogenicity, were excluded from the study. Thus, five compounds without any RO5 and other toxicities were selected for further evaluation.

MD simulation analysis through RMSD, RMSF, SSE and ligand property plots revealed three compounds out of five (secoisolariciresinol, syringaresinol and cleomiscosin A) with stable structure conformations that were subjected to further in silico and in vitro enzymatic assay, MTT cytotoxicity and apoptosis analysis.

In the current study, we also investigated the inhibition of 6-PGD by syringaresinol and cleomiscosin A using an in vitro enzymatic assay by using purified 6-PGD from human erythrocytes and A546 lung cancer cells lysate protein. The results of our study demonstrated that both lignan compounds acted as inhibitors against 6-PGD.

Fortunately, all three final hits belong to class lignans that have been reported previously as a diversified source of drugs with anticancer potentiality [56]. Syringaresinol, a lignan compound, has been found to be naturally constituted in different plants, for instance, *Magnolia thailandica, Panax ginseng* berries and *Prunus mume* [57], while the presence of the second lignan, cleomiscosin A, has been reported in several plant resources such as *Rhododendron collettianum Cleome viscosa*, *Acer nikoense*, *Hyoscyamus niger*, etc. [58]. In silico cytotoxicity evaluation showed the inhibitory potential of compounds in terms of their activation and inactivation probability. Compound syringaresinol showed higher activation potential, while cleomiscosin A trailed the earlier compound against A549 cells, and secoisolariciresinol showed less activation probability in comparison to the former, with a cut-off value of Pa > Pi.

The anticancer potential of syringaresinol against different cell lines, such as A549 and MCF-7 with IC_50_ ≥ 100 μM [59] and Hela, Hep-2 and C6 with IC_50_ 31.7, 28.0 and 10.7 μg/mL, respectively, has already been reported [60]. Several other scientific studies have also reported the cytotoxicity of cleomiscosin A for lung (A549), breast (MCF-7) and colon (PC-3) cancers [61], showing IC_50_ values of 130.8, 132.1 and 130.1 μg/mL, respectively. The IC_50_ values of our studies for both compounds syringaresinol (IC_50_ = 36.9 μg/mL) and cleomiscosin A (IC_50_ = 133 μg/mL) against lung cancer (A549) cells are parallel with earlier reported data.

Apoptosis is a systematic way of suicidal cell death that is efficiently controlled at the gene level, resulting in efficient and organized removal of defective cells during developmental and DNA damage processes [62]. Cancer prevention is attributed as a key function of apoptosis [63]. Syringaresinol has been reported earlier for inhibiting HL-60 cell proliferation via cell cycle arrest in the G_1_ phase and apoptosis induction [64]. In the present study, flow cytometric analysis investigated that both compounds syringaresinol and cleomiscosin A inhibited cell growth in A549 cells by inducing apoptosis. The results found that syringaresinol and cleomiscosin A induced significant apoptosis. The rate of apoptosis increased to 28.3, 37.3, 42.1 and 46.2% for syringaresinol and 17.3, 28.3, 34.8 and 42.6% for cleomiscosin A at selected concentrations in comparison to control cells.

## 5. Conclusions

Virtual screenings are highly coveted due to fewer time and resource requirements to search small molecule databases in the field of drug discovery. Following this idea, in the current study, molecular docking was performed using a library of 17,000 phytochemicals against 6-PGD, which exhibited 7 potential ligands with satisfying binding ability to essential residues in the substrate binding cavity. Furthermore, ADMET and toxicity drug scan exploration indicated five compounds with good drug-likeness and toxicity profiles. In addition, MD simulation study revealed three top-ranking hits (secoisolariciresinol, syringaresinol and cleomiscosin A) with stable conformations. Interestingly, all three obtained hits were found to be members of the same class of phytochemicals, the lignans. The in vitro enzymatic assay validated the inhibitors’ (syringaresinol and cleomiscosin A) inhibition against 6-PGD. Moreover, cytotoxicity and apoptosis studies were carried out to evaluate the lignans’ potential to inhibit the growth of A549 cells and their mode of cell death, which observed syringaresinol and cleomiscosin A as potent anti-lung cancer agents by inducing apoptosis. On the basis of the results obtained during the present study, the lignans syringaresinol and cleomiscosin A can be remarked as natural anti-lung cancer metabolic modulators with acceptable drugability properties against 6-PGD. However, further in vitro (NADPH and protein binding assays), in vivo and clinical trials are recommended to elucidate the underlying mechanism for future studies.

## Figures and Tables

**Table 1:** Potential hits summary: their binding score, RMSDs, residual interaction and H-bonds.

Name	CID	Score	RMSD	Binding Site Residual Interaction	Hydrogen Bonds
Secoisolariciresinol	65,373	−12.9507	1.4987	Arg288, Lys184, Gly130, Ser129	3
Syringaresinol	100,067	−10.3479	1.6195	Tyr192, Arg288, Lys184, Ala258, Gly130	5
Cleomiscosin A	442,510	−10.3156	1.0467	Arg288, Glu191, Lys184	4
Tubulosine	72,341	−10.0725	1.7187	Tyr192, Lys184	2
Terrestriamide	5,321,824	−10.0456	1.7236	Arg288, Lys184	2
3-Phosphoglyceric acid	Natural inhibitor	−8.4342	1.3230	Tyr192, Arg288, Glu191	3

CID: compound ID; RMSD: root mean square deviation.

**Table 2:** Results of compounds examined for Lipinski’s rule.

Compounds	Lipinski’s RO5						
	Molecular Weight (g/mol)	Number of HBA	Number of HBD	MlogP	Veber’s Rule		Violations
					TPSA (Å)	RB	
Syringaresinol	418	8	2	2.62	95.86	6	0
Cleomiscosin A	386.3	8	2	1.96	107.60	4	0
Secoisolariciresinol	362.4	6	4	2.8	99.3	9	0
Terrestriamide	327.3	6	3	1.78	95.6	6	0
Tubulosine	475.6	6	3	4.86	69.8	5	0

HBA: hydrogen bond acceptor; HBD: hydrogen bond donor; MlogP: moriguchi octanol water partition coefficient.

**Table 3:** ADMET profiles of shortlisted phytochemicals.

Compounds					
Parameters	Syringaresinol	Tubulosine	Secoisolariciresinol	Terrestriamide	Cleomiscosin A
Absorption					
GI absorption	High	High	High	High	High
Water solubility (log mol/L)	−3.92	−3.41	−3.76	−3.13	−3.64
Skin permeability (cm/s)	−7.27	−5.93	−6.72	−6.65	−7.15
P-gp substrate	No	Yes	No	No	No
P-gp inhibitor	Yes	Yes	No	No	Yes
Bioavailability score	0.55	0.55	0.55	0.55	0.55
Distribution					
BBB	No	Yes	No	No	No
Subcellular localization	Mitochondria	Mitochondria	Mitochondria	Mitochondria	Mitochondria
Metabolism					
CYP1A2 inhibitor	No	No	Yes	No	No
CYP2C19 inhibitor	Yes	No	Yes	No	No
CYP2C9 inhibitor	Yes	No	No	No	No
CYP2D6 inhibitor	No	Yes	No	No	No
CYP3A4 inhibitor	Yes	No	No	Yes	No
Excretion					
Total clearance (log mL/min/kg)	0.255	1.082	0.248	0.211	0.394
Toxicity					
Carcinogenicity	No	No	No	No	No
Toxicity [36]	No	No	No	No	No
Hepatotoxicity	No	No	No	No	No
Oral rat acute toxicity (LD50 mol/kg)	2.59	2.76	2.03	2.11	2.7031
Synthetic accessibility	4.36	4.95	3.21	2.55	4.47

GI: gastrointestinal; BBB: blood–brain barrier; P-gp: P-glycoprotein; LD50: lethal dose; CYP: cytochrome p450.

**Table 4:** In silico cytotoxicity prediction of selected hits.

Compounds	Cancer Cell Line	Pa	Pi	Tumor Type
Syringaresinol	HOP-18	0.559	0.006	Non-small-cell lung carcinoma
	PC-6	0.549	0.020	---
	A549	0.561	0.047	Small epithelial cell lung carcinoma
	NCI-H187	0.434	0.043	Non-small-cell lung carcinoma
	MDA-MB-453	0.426	0.053	Breast adenocarcinoma
	NCI-H838	0.452	0.083	Small cell lung carcinoma
	HCC-2998	0.404	0.035	Colon adenocarcinoma
	OVCAR-4	0.407	0.046	Ovarian adenocarcinoma
	H9	0.358	0.030	Leukemia
	NALM-6	0.383	0.061	Leukemia
	DMS-114	0.421	0.104	---
	CFPAC-1	0.357	0.071	Pancreatic carcinoma
	NCI-H322M	0.349	0.067	Non-small-cell lung carcinoma
	OVCAR-5	0.347	0.067	Ovarian adenocarcinoma
	MKN-7	0.301	0.047	Gastric carcinoma
	HCT-15	0.311	0.068	Colon adenocarcinoma
	HL-60	0.303	0.085	Leukemia
Cleomiscosin A	HL-60	0.642	0.013	Leukemia
	MCF-7	0.567	0.038	Breast carcinoma
	PC-6	0.407	0.039	Non-small-cell lung carcinoma
	NALM-6	0.388	0.051	Leukemia
	A549	0.358	0.103	Small epithelial cell lung carcinoma
Secoisolariciresinol	MDA-MB-453	0.443	0.033	Breast adenocarcinoma
	PC-6	0.389	0.044	Small cell lung carcinoma
	HOP-18	0.339	0.053	Non-small-cell lung carcinoma
	HCT-15	0.341	0.056	Adenocarcinoma
	SK-MEL-2	0.306	0.078	Melanoma
	A549	0.250	0.166	Small epithelial cell lung carcinoma

Pa > 0.3, Pa: probability to be active; Pi: probability to be inactive.

## Data Availability

The authors may be contacted for more details concerning data supporting the reported results. The data used to support the findings of this study are included within the article.
